# Research on the Equity and Influencing Factors of Medical and Health Resources Allocation in the Context of COVID-19: A Case of Taiyuan, China

**DOI:** 10.3390/healthcare10071319

**Published:** 2022-07-16

**Authors:** Xueling Wu, Ye Zhang, Xiaojia Guo

**Affiliations:** School of Geographical Sciences, Shanxi Normal University, Taiyuan 030031, China; wuxueling@sxnu.edu.cn (X.W.); 220113019@sxnu.edu.cn (Y.Z.)

**Keywords:** medical and health resources allocation, equity, Theil index, agglomeration degree, grey relational analysis, Taiyuan

## Abstract

COVID-19 has killed millions of people worldwide. As a result, medical and health resources continue to be strained, posing a great threat to people’s safety and economic and social development. This paper built the index system of influencing factors of medical and health resources containing the economy, population and society, and then classified Taiyuan into three types of regions by cluster analysis. The Gini coefficient, Theil index and agglomeration degree were then used to analyze the spatial distribution of medical and health resources allocation, and its influencing factors were studied by grey relational analysis. It was found that the population allocation of medical and health resources in Taiyuan was better than area allocation. Population has the greatest influence on the allocation of medical and health resources, followed by society and the economy. The more developed the regional economy, the more diversified the main influencing factors, and the more adjustment and control choices of medical and health resources allocation. Suggestions for optimal allocation were put forward in order to fully utilize the limited medical and health resources, effectively respond to the epidemic needs, promote the sustainable development of resources, protect the health of residents, and improve social benefits.

## 1. Introduction

The theme of the United Nations (UN) 2030 sustainable development agenda is the security and well-being of all mankind, and global health security is also a prerequisite and key symbol for the realization of this goal [1]. However, in recent years, natural disasters caused by climate change and various epidemics have exerted a great impact on global development, and have also sent a warning to human society to achieve the United Nations 2030 Sustainable Development Goals (SDGs) as scheduled [2]. In particular, COVID-19, which has killed millions of people across 215 countries, poses a huge threat to the health of people around the world [3,4,5]. The impact of the COVID-19 on the economic order and social activities has been unprecedented. All trades and professions have been seriously damaged, and the most direct impact is naturally the medical and health undertakings in various countries. China has a large population and frequent population migration. The suddenness, high infectivity and huge destructiveness of COVID-19 suggest that we must be vigilant against the epidemic. Existing studies show that people’s sense of security in public emergencies is usually an intuitive dimension to measure the degree of national modernization [6]. However, China has always adhered to the dynamic zero-COVID policy and people’s life first. The multipoint continuous outbreak of the epidemic has aggravated tension concerning the emergency of medical and health resources in various parts of China, which has had an effect on people’s health. Although the “Healthy China” strategy has led to the rapid growth of medical and health resources in China, there are still problems such as difficult and expensive medical treatment for ordinary people due to the imbalance between supply and demand of medical and health resources. Therefore, during the COVID-19 pandemic, it remains a serious challenge for China to rationalize and optimize the allocation of medical and health resources in order to safeguard the original treatment of chronic diseases while responding in a timely manner to the large number of confirmed patients and asymptomatic infected patients.

Foreign scholars have used statistical models to conduct empirical analyses on medical and health resources allocation [7,8,9,10,11]. The Gini coefficient was used to study medical and health resources allocation in the 1980s [12,13,14], and has been widely applied in the distribution of medical and health resources since then [15,16,17,18,19,20]. Limited to reflecting only the overall distribution, the Gini coefficient is often used in conjunction with other methods [21]. Among them, it is widely used in combination with regression models, while the index of dissimilarity, the Theil index and other methods [22,23,24,25], the Delphi survey method [26], the concentration index [27] and ArcGIS are also used in medical and health resources allocation. Research results show that the inequitable allocation of medical and health resources is common in all countries of the world, and inequity is mainly reflected the differences between regions and within regions, as well as in differences in human, material and financial resources [28,29]. There are many factors that cause the unequal allocation of medical and health resources, including system, market, economy, medical insurance and so on [30,31,32,33,34,35]. According to Shi Baoguo, China’s elite hospitals have evident spatial heterogeneity, and the city level is the most important factor affecting the spatial allocation. Its action intensity shows a solid and weak mosaic trend in the Middle East, relatively concentrated in some areas with medium intensity, and concentrated in the West China [35].

In the early 21st century, Chinese scholars began to quantitatively study the allocation of medical and health resources by using the Lorenz curve, Gini coefficient, concentration index, Theil index and other methods, and the research scope was mostly at the national and provincial scale [36,37] and less involved at the municipal and below [38]. The findings show that the inequitable allocation of medical and health resources exists at all scales studied, with geographic allocation being less equitable and differences in medical and health resources allocation being caused between regions and within regional differences [39,40,41,42,43,44]. Affected by the economy, population, society and policy, the allocation of medical and health resources varies greatly among regions [45]. The influence of various factors on medical and health resources allocation among different regions, global and local factors should be judged reasonably and resources allocation strategies should be tailored to local conditions [46].

In addition, Taiyuan is a typical mountainous city, with mountainous and hilly terrain accounting for about 80% of the total area. Geographical accessibility in some areas cannot be met by population allocation alone, and population allocation and area allocation need to be considered together. Furthermore, according to the results of a study by Song Xueqian and Zhang Ye et al., the results of choosing population allocation and geographical allocation have some deviations in reflecting the spatially balanced characteristics of health care resources due to the differences in population size and area of administrative districts, and the importance of geographical equity should be increased [46,47].

In view of this, this paper combines geographic spatial analysis in the context of COVID-19, further refines the study scale, takes the county as the basic research unit, combines both demographic and geographical factors to allocate resources, and uses cluster analysis to divide Taiyuan into three types of regions with respect to the current spatial allocation of medical and health resources in Taiyuan. The combination methodology of Gini coefficient, Thiel index, entropy method, agglomeration degree and grey relational analysis were used to analyze the characteristics of the spatial allocation of medical and health resources in Taiyuan, to examine the equity of the allocation of medical and health resources and its influencing mechanism, and then to propose strategies for the optimal spatial allocation of medical and health resources in accordance with the objectives of the Healthy Shanxi 2030 Plan. The study of the complex system of medical and health system through the interdisciplinary model of geography and public health is not only of great scientific value, but also of great practical significance in guiding the scientific decisions of the medical and health service system, meeting public demand for medical and health services and effectively responding to the needs of public health emergencies such as COVID-19.

## 2. Materials and Methods

### 2.1. Study Area

Taiyuan is located in the middle of Shanxi Province, with coordinates of 111°30′–113°09′ E and 37°27′–38°25′ N, lying tilted from north to south, west, north and east. It is surrounded by mountains, and a plain in the center and southern areas. As the provincial capital, Taiyuan is the political, economic and cultural center of Shanxi, with a resident population of 5,390,957 at the end of 2021 and an area of 6999 square kilometers. The whole city includes six districts, Xiaodian, Yingze, Xinghualing, Jiancaoping, Wanbailin, Jinyuan, three counties: Qingxu, Yangqu and Loufan, and one county-level city, Gujiao. In 2021, the GDP of Taiyuan City was 5121.61 billion yuan, among which the GDP of Yingze, Xiaodian and Xinghualing were all above 80 billion yuan, showing a high level of economic development, while Qingxu, Jinyuan, Gujiao, Yangqu and Loufan were at less than 30 billion yuan.

### 2.2. Methods

#### 2.2.1. Gini Coefficient

The Gini coefficient ranges from 0 to 1. A smaller value indicates a fairer distribution. Generally, 0.4 is regarded as the warning line, below 0.2 is absolutely equity, and above 0.5 is a wide gap [41]. The calculation formula is:

$$G=1-\overset{n}{\underset{i=1}{\sum }}(X_{i}-X_{i-1})\left(Y_{i}+Y_{i-1}\right)$$

where *n* is the total number of districts and counties in Taiyuan, *n* = 10, *i* is the ascending order of medical and health resources in districts and counties, *X_i_* is the cumulative percentage of the population of districts and counties, *Y_i_* is the cumulative percentage of medical and health resources, *X*_0_ = 0, *Y*_0_ = 0.

#### 2.2.2. Theil Index

The Theil index was the earliest indicator to measure the income gap. Because it makes up for the defect of Gini coefficient whereby it cannot analyze the differences between regions and within regions, it has been used in the research on equity of medical and health resources allocation in recent years. The smaller the Theil index, the fairer the allocation. The calculation formula is:

$$T=\overset{n}{\underset{i=1}{\sum }}P_{i}\log\frac{P_{i}}{Y_{i}}$$

where *T* is the Theil index of the allocation of medical and health resources in Taiyuan, *P_i_* is the proportion of population (area) of district and county *i* in the total population (area) of Taiyuan, *Y_i_* is the proportion of the quantity of medical and health resources of district and county *i* in the total medical and health resources of Taiyuan. The Theil index can be decomposed into:

$$T=T_{W}+T_{A}$$

$$T_{W}=\overset{k}{\underset{g=1}{\sum }}P_{g}T_{g}$$

$$T_{A}=\overset{k}{\underset{g=1}{\sum }}P_{g}\log\frac{P_{g}}{Y_{g}}$$

*T_W_* and *T_A_* are the differences within and among categories in the allocation of medical and health resources in the three regions of Taiyuan, respectively; *k* is the number of categories in Taiyuan, *k* = 3; *P_g_* is the proportion of population in category *g*, *g* = 1, 2, 3, *Y_g_* is the proportion of the total medical and health resources in category *g*; *T_g_* is the Theil index for category *g*.

The Theil index can measure the contribution of differences in medical and health resources allocation within and among categories to the total Theil index, which are expressed by *C_W_* and *C_A_* respectively:

$$C_{W}=\frac{T_{W}}{T}$$

$$C_{A}=\frac{T_{A}}{T}$$

#### 2.2.3. Agglomeration Degree

The allocation of medical and health resources is often evaluated through the combined analysis of the two indicators, Health Resources Agglomeration Degree (HRAD) and Population Agglomeration Degree (PAD) [42]. The HRAD indicates the area allocation of medical and health resources, that is, the ratio of medical and health resources distributed per unit area of districts and counties to that of the whole of Taiyuan. HRAD = 1, indicating that the resources allocation of districts and counties is equal to the Taiyuan average, and the area allocation is absolutely equity; HRAD < 1, indicates that the sparse resources allocation of districts and counties is lower than the average; HRAD >1, indicating that resources allocation of districts and counties level is above-average. PAD refers to the proportion of population density of the county to that of the whole of Taiyuan, reflecting the relative concentration of population within a region [38]. The ratio of HRAD to PAD represents the level of medical and health resources allocated by population, that is, the ratio of medical and health resources per capita in districts and counties to that of the whole Taiyuan. If the ratio is less than 1, it means that the county resources are sparsely allocated per capita, and vice versa [42].

$${\mathrm{HRAD}}_{i}=\frac{{\mathrm{HR}}_{i}/{\mathrm{HR}}_{n}}{A_{i}/A_{n}}$$

$${\mathrm{PAD}}_{i}=\frac{P_{i}/P_{n}}{A_{i}/A_{n}}$$

$$\frac{{\mathrm{HRAD}}_{i}}{{\mathrm{PAD}}_{i}}=\frac{{\mathrm{HR}}_{i}/{\mathrm{HR}}_{n}}{P_{i}/P_{n}}$$

HR*_i_* and HR*_n_* are the amount of medical and health resources in district and county *i* and Taiyuan respectively, *A_i_* and *P_i_* are the area and population of district and county *i*, *A_n_* and *P_n_* are the area and population of Taiyuan.

#### 2.2.4. Grey Relational Analysis

Grey relational analysis is a method to judge the correlation according to the geometric shape similarity between the index sequence curve and the selected index sequence curve. Grey relational degree indicates the contribution of each factor to the system. The greater the grey relational degree, the stronger the correlation. The calculation steps are as follows: $X_{0}^{\prime }$ and $X_{i}^{\prime }$ are obtained by the dimensionless processing of data using the initial value method, and the absolute value of the difference sequence $|X_{0}^{\prime }-X_{i}^{\prime }|$, the relational coefficient $\xi_{0i}\left(k\right)=\frac{△min+\rho △max}{△oi\left(k\right)+\rho △max}$, and the grey relational degree $\gamma_{i}\left(k\right)=\frac{1}{N}\overset{N}{\underset{k=1}{\sum }}\xi 0i\left(k\right)$ are calculated. ρ represents the resolution coefficient, which is 0.5 in this paper. This paper constructs the index system of allocation influencing factors from three aspects, the economy, population and society (Table 1).

### 2.3. Data Source

The data of this study comes from the *Taiyuan Statistical Yearbook* and the *Shanxi Statistical Yearbook* from 2004 to 2020, the *China Population Census County Data by counties* in 2000, 2010 and 2020, and the seventh census of Taiyuan. The missing data for individual years were replaced by the linear trend method of adjacent points. The population allocation of medical and health resources in this study is the number of medical and health resources per thousand population, and the area allocation is the number of medical and health resources per square kilometer.

## 3. Results

### 3.1. Equity of Medical and Health Resources Allocation in Taiyuan

According to the number of beds in medical and health institutions, the number of health technicians and the number of licensed (assistant) physicians in Taiyuan in 2019, the k-means cluster analysis was carried out for districts and counties in Taiyuan, and it was classified into three types of regions according to the descending order of medical and health resources allocation. Class Ⅰ includes Yingze and Xinghualing; class Ⅱ includes Xiaodian, Jiancaoping and Wanbailin; and class Ⅲ includes Jinyuan, Loufan, Yangqu, Qingxu and Gujiao.

#### 3.1.1. Equity Based on Gini Coefficient

From 2003 to 2019, the Gini coefficient of medical and health resources allocated by population in Taiyuan decreased slightly, but they were all less than 0.3, indicating relatively high equity. The Gini coefficient of beds ranged from 0.2 to 0.3, which was relatively equal. The Gini coefficient of health technicians and licensed (assistant) physicians fluctuated, which was absolutely equity after 2016. Among them, licensed (assistant) physicians were better allocated by population. The Gini coefficients based on area allocation of medical and health resources in Taiyuan were all greater than 0.8, with a wide gap and a high inequity. The Gini coefficients of each index increased slightly and the equity decreased slightly (Table 2).

It is worth noting that, taking 2010 as the turning point, the Gini coefficient based on population allocation showed a trend of increasing first and then decreasing, indicating the improvement in resources allocation equity after 2010. This rule does not apply to area allocation, although their Gini coefficients were higher.

#### 3.1.2. Equity Based on Theil Index

From 2003 to 2019, the Theil index of medical and health resources in Taiyuan varied from 0.0413 and 0.7166, with significant difference. The Theil index of population allocation and that of area allocation showed a general upward trend, and the inequity increased. The Theil index based on population allocation was less than that of area allocation, and the growth rate of the former was higher, indicating that population allocation was better than area allocation, and the trend of inequality expansion in population allocation is more obvious. The *C_A_* of each indicator is higher than that of *C_W_*, indicating that inter-regional difference is the main aspect of allocation inequity (Table 3).

The Theil index based on population allocation was less than 0.1 and relatively equity. Among them, the Theil index of health technicians was between 0.06 and 0.07, and the equity was the weakest. The *T_W_* and *C_W_* of each index decreased, while the *T_A_* and *C_A_* increased, indicating that the allocation of medical and health resources in the region tended to be equity, while the inter-regional differences further increased, and its influence on regional differences was growing.

The Theil index based on area allocation ranged from 0.5750 to 0.7166, with weak equity. Health technicians remained the weakest, with a Theil index between 0.6 and 0.7. The *T_W_* and *T_A_* of each medical and health resources increased in general, but the *T_W_* of beds decreased, which further indicated that the poor equity of area allocation of medical and health resources. In 2019, the Theil index of bed (0.6725) exceeded that of licensed (assistant) physicians (0.6622), indicating that the area allocation of the highly skilled has received more attention and has improved greatly.

#### 3.1.3. Equity Based on Agglomeration Degree

**Figure 1:**
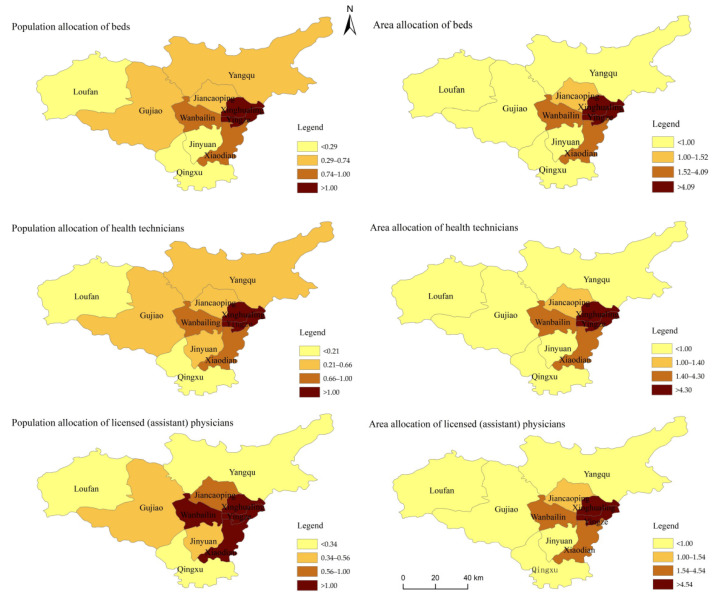
Agglomeration of medical and health resources in Taiyuan in 2019.

Following the pattern of previous years, in 2019, the highly agglomeration of area allocation was semi-circular, concentrated the central and eastern Taiyuan with good economic development. Among them, the agglomeration degrees of Yingze and Xinghualing were significantly higher than that of Xiaodian and Wanbailin, which were higher than that of Jiancaoping. Based on population allocation, beds and health technicians were concentrated in Yingze and Xinghualing in central and eastern Taiyuan, while licensed (assistant) physicians were concentrated in Yingze, Xinghualing, Xiaodian and Wanbailin, showing a semi-circular distribution (Figure 1).

Based on area allocation, the HRADs in class Ⅰ and class Ⅱ were greater than 1. The medical and health resources were mainly distributed in class, with HRAD above 9, and the allocation of resources were rich. The HRAD of class Ⅱ were between 1.40 and 4.54, which was closest to 1, with the best equity in the three types of regions. The HRAD of class Ⅲ was mostly less than 1, indicating that the medical and health resources were scarce based on area allocation. Loufan had the lowest HRAD of 0.034, and the inequity needed to be improved urgently. From 2003 to 2019, the HRAD in class Ⅲ declined with fluctuation, while Yingze in class Ⅰ and Xiaodian in class Ⅱ wavelike rose, and the inter-regional inequality was further increased.

Based on population allocation, the HRAD/PAD of class Ⅰ was between 1.45 and 1.97, with an obvious concentration advantage. In class Ⅱ, the HRAD/PAD of Wanbailin was nearest to 1, and the equity was the best, while Xiaodian and Jiancaoping were around 0.7. The HRAD/PAD of Class Ⅲ were mostly less than 1 and mostly around 0.4, especially Qingxu and Loufan. See Table A1 in Appendix A.

#### 3.1.4. Comprehensive Evaluation of Medical and Health Resources Allocation in Taiyuan

The weighting of the evaluation index in entropy method was confirmed, as far as possible, to remove evaluation subjective factors. The linear weighting used to obtain the evaluation values of medical and health resources in districts and counties was based on population allocation, area allocation, and comprehensive allocation, and were obtained by linear weighting index. There are significant differences among the three types of regions. Allocation in class Ⅰ was higher than that in class Ⅱ, which was higher than that in class Ⅲ.

**Figure 2:**
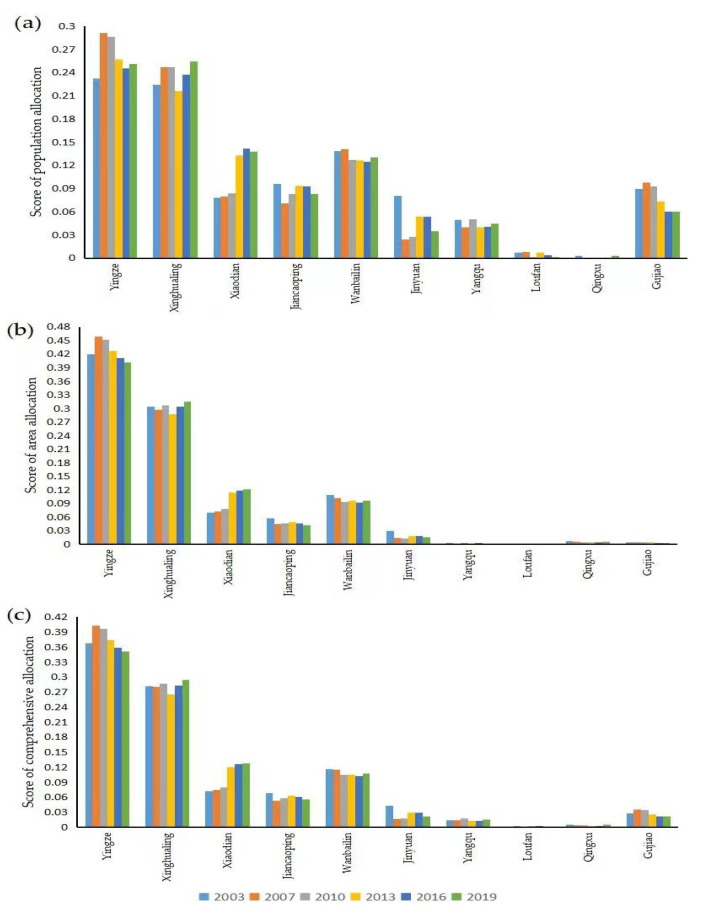
Score of districts and counties in Taiyuan, 2003–2019. (**a**): Population allocation; (**b**): Area allocation; (**c**): Comprehensive allocation.

There are significant differences in population allocation of medical and health resources among regions. The score of class Ⅰ was about 0.25, which was relatively high. It was about 0.1 in class Ⅱ, and was less than 0.1 in class Ⅲ. The population allocation in Yingze was the highest, and that in Qingxu was the lowest, with a difference of about 0.29. The population allocation of Yingze, Xinghualing and Xiaodian fluctuated rose. Among them, Xiaodian grew, increasing by 0.06, with the largest fluctuation from 2003 to 2019. The population allocation of other districts and counties declined with fluctuation, among which Jinyuan and Gujiao showed a significant downward trend, decreasing by 0.05 and 0.03, respectively. See Figure 2a.

The area allocation was significantly different among districts and counties. Yingze has the highest score and Loufan has the lowest, with a difference of about 0.5. The area allocation of Xinghualing, Xiaodian and Loufan showed an upward trend, among which the growth rate of Xiaodian reached 74%. Other districts and counties declined with fluctuation. See Figure 2b.

The comprehensive allocation score of class Ⅰ was about 0.4, which was relatively high. The score of class Ⅱ was about 0.1, and class Ⅲ was lower than 0.04. Yingze had the highest score and Loufan had the lowest score, with an obvious difference of about 0.4. The comprehensive allocation of Xinghualing, Xiaodian and Yangqu increased, especially in Xiaodian. The other counties fluctuated and declined, among which Loufan and Jinyuan declined greatly. See Figure 2c.

### 3.2. Influencing Factors and Mechanism of Medical and Health Resources Allocation

The grey relational degrees of the influencing factors selected in this study were all greater than 0.5, indicating that there was a great correlation between the reference series and the comparison series, reflecting that the influencing factors selected in this study were relatively scientific.

#### 3.2.1. Economy

Economic development is the guarantee for the development of medical and health resources. The proportion of secondary industry (*X*_3_) and tertiary industry (*X*_4_) had great influence on the allocation of medical and health resources in various regions, and the relational degree were more than 0.8. As a coal resources city, Taiyuan’s secondary industry occupies an important position in the economic structure, which has a great influence on the allocation of medical and health resources. With the upgrading of industrial structure, the tertiary industry has played a prominent role and the supply capacity of medical and health resources has been further enhanced.

The influence of other economic indicators on the allocation of medical and health resources in class Ⅲ (about 0.8) was higher than that in class Ⅰ and class Ⅱ (both about 0.7). Because of the weak economic foundation and low income, the improvement of economy and income has a more obvious driving effect on the medical and health resources. Therefore, GDP (*X*_1_), per capital GDP (*X*_2_), per capita disposable income of urban residents (*X*_5_) and per capita disposable income of rural residents (*X*_6_) have greater influence on class Ⅲ.

#### 3.2.2. Population

Population is the dominant factor affecting the allocation of medical and health resources [46]. The relational degrees between the allocation of medical and health resources and indicators of population in various regions were above 0.9.

Based on the people-oriented principle, total population (*X*_7_) is the main basis for allocating medical and health resources. Highly educated people have strong awareness of disease prevention and higher income, and are also able to invest in health. Given their weak constitution, the elderly and children have a great demand for medical and health resources. Therefore, the proportion of aging (*X*_9_) and the proportion of children (*X*_10_) have important effects on the allocation of medical and health resources.

#### 3.2.3. Society

The urbanization rate (*X*_11_), fiscal expenditure decentralization (*X*_13_), fiscal revenue decentralization (*X*_14_) and highway mileage (*X*_16_) have important impacts on the allocation of medical and health resources in each region, and the relational degrees were about 0.9. The relational degree of total retail sales of consumer goods in class Ⅲ was about 0.7, which is higher than that in class Ⅰ and class Ⅱ (0.537–0.610), while the relational degree of number of participants in basic medical insurance (0.7) is significantly lower than that in class Ⅰ and class Ⅱ (0.9). See Table A2 in Appendix A.

Urbanization not only promotes the development of public service facilities, but also attracts population agglomeration, which further increases the demand for medical and health resources, forming a snowball effect and promoting the improvement of allocation of medical and health resources. The total retail sales of consumer goods reflect the purchasing power of social goods. As a rigid demand, medical and health resources are less affected by the purchasing power of residents, but the relational degree is slightly higher in class Ⅲ, which has limited purchasing power. Fiscal expenditure decentralization reflects the investment opportunity for medical and health resources, while fiscal revenue reflects the local financing capacity and resource investment capacity, so the allocation of medical and health resources is greatly affected by finance. Basic medical insurance can reduce medical costs and stimulate medical demand. Therefore, the number of basic medical insurance insured is highly correlated in class Ⅰ and class Ⅱ. The relational degree of this index is low in class Ⅲ, which may be related to the fact that the medical and health resources are relatively backward, and the residents tend to medical treatment in different places with reduced medical insurance benefits. Highway mileage reflects the accessibility of medical and health resources and has a positive impact on its allocation equity.

## 4. Discussion

COVID-19 has entered the stage of normalization prevention and control. Taiyuan will face the problems of medical and health resources run and bed shortage. Population allocation and area allocation are two aspects of equity of medical and health resources allocation which reflect its shortcomings from different perspectives. Population allocation of medical and health resources was better than area allocation in Taiyuan. Due to the regional differences and the tradition of allocating medical and health resources according to population, the allocation of resources and facilities was relatively scarce in areas with sparse population and inconvenient transportation, resulting in unfair area allocation. Other scholars reached similar conclusions [48,49,50]. Based on population allocation, the equity of beds and licensed (assistant) physicians improved gradually, while that of health technicians decreased. Based on area allocation, all health resources were in extremely unfairness and the unfairness was further increasing. To strengthen the focus on the equity on area allocation, improve the medical and health resources allocation in areas with sparsely population and weak economy, especially in rural areas, and reduce the urban-rural gap is not only the need to create the ‘15-min health care service circle’, but also the path to achieve regional health security and enhance the security of people during the epidemic. In order to ensure the needs of epidemic prevention, it is necessary to establish designated hospitals, increase the allocation of beds and medical staff, and improve the prevention and control of the epidemic.

According to the allocation of medical and health resources, Taiyuan was divided into class Ⅰ (Yingze, Xinghualing), class Ⅱ (Xiaodian, Jiancaoping, Wanbailin) and class Ⅲ (Jinyuan, Loufan, Yangqu, Qingxu, Gujiao), in descending order, and their comprehensive level of medical and health resources were evaluated. From 2003 to 2019, the comprehensive allocation in all districts and counties descended, except for Xiaodian and Xinghualing, with great differences among them. Inter-regional differences were the main aspects of differences in population allocation and area allocation.

Different from Xinjiang [47] and Shandong [51], and similar to Sichuan [46], population had the greatest influence on the allocation of medical and health resources, followed by society and economy in Taiyuan. In addition, there were spatial scale differences in the influencing factors. Economy, population, finance and transportation had great effects on class Ⅰ. The influencing factors in class Ⅱ were mainly the proportion of secondary industry, population and finance, while those in class Ⅲ were mainly the proportion of secondary production and population structure. Therefore, it is necessary to adjust and control the allocation of medical and health resources in the three regions according to local conditions. Class Ⅰ should improve the quality of medical and health resources allocation, improve the level of education, regulate the population structure, take the new road of intensive, green, low-carbon urbanization, and improve transportation so as to optimize the allocation. Class Ⅱ should improve the elderly security system, increase the government health investment, and expand the financial sources. And class Ⅲ should increase the allocation of medical and health resources, achieve the balance between supply and demand, attract technical talents, promote the development of the medical community, as well as improving the elderly security system.

In addition, the COVID-19 has entered a stage of normalization prevention and control, and Taiyuan City has problems such as medical and health resources usage and bed shortages. In order to ensure the needs of COVID-19 prevention and control, effectively prevent, timely control and eliminate the hazards of public health emergencies, protect public health and life safety, and maintain normal social order. It is necessary to set up designated hospitals to undertake the task of preventing and controlling infectious diseases, increase the allocation of beds and medical staff, and improve the prevention and control of the epidemic.

## 5. Conclusions

The allocation of medical and health resources in Taiyuan was relatively equity based on population, but highly inequitable based on area. The comprehensive allocation of districts and counties tended to decrease. Among the three regions, or among districts and counties, there were great differences in the allocation, and inter-regional differences were the main aspects of regional differences. There were significant regional differences and spatial scale differences in the influencing factors of medical and health resources allocation. Population was the most important factor affecting the allocation of medical and health resources in Taiyuan. There were great differences among the influential factors in the three regions of Taiyuan. Regions with developed economies had diversified influencing factors and a wider range of choices and stronger maneuverability in regulatory policies of medical and health resources allocation.

The current statistical data on medical and health resources in Taiyuan cannot fully meet the research demand, and it is difficult to obtain data at the sub-county scale. In the future, data can be obtained through field research and other means to refine the research scale to the village and township level in order to respond to the rural revitalization strategy and improve the current situation of medical and health resources allocation more accurately and purposefully. The influencing factors study found that the more economically developed the region, the more diverse the influencing factors, and the wider the choice of medical and health resources allocation regulation and control direction, the more operable it is. The extent to which economic development will cause a qualitative change in medical and health resources is a direction for further research in this field.

## Figures and Tables

**Table 1:** Index system of influencing factors of medical and health resources allocation in Taiyuan.

System	Number	Index	Unit	Description
Economy	X_1_	GDP	million yuan	
	X_2_	per capital GDP	million yuan	
	X_3_	proportion of secondary industry	%	
	X_4_	proportion of tertiary industry	%	
	X_5_	urban per capita disposable income	yuan	
	X_6_	per capita disposable income of rural residents	yuan	
Population	X_7_	total population	million people	
	X_8_	education degree	%	proportion of primary and secondary school students in the total population
	X_9_	proportion of ageing	%	proportion of the population aged 65 and over to total population
	X_10_	proportion of children	%	proportion of population under 14 to total population
Society	X_11_	urbanization rate	%	proportion of urban population in total population
	X_12_	total retail sales of consumer goods	million yuan	
	X_13_	fiscal expenditure decentralization	%	proportion of county financial expenditure in Taiyuan financial expenditure
	X_14_	fiscal revenue decentralization	%	proportion of county financial revenue in Taiyuan financial revenue
	X_15_	number of participants in basic medical insurance	people	
	X_16_	highway mileage	kilometer	

**Table 2:** Gini coefficient of medical and health resources in Taiyuan, 2003–2019.

	Population Allocation			Area Allocation		
	Beds	Health Technicians	Licensed (Assistant) Physicians	Beds	Health Technicians	Licensed (Assistant) Physicians
2003	0.258	0.192	0.180	0.896	0.907	0.905
2007	0.262	0.245	0.205	0.915	0.917	0.912
2010	0.289	0.269	0.227	0.914	0.920	0.912
2013	0.224	0.203	0.194	0.906	0.911	0.909
2016	0.229	0.196	0.166	0.908	0.913	0.908
2019	0.246	0.198	0.160	0.912	0.912	0.906

**Table 3:** Theil index of medical and health resources allocation in Taiyuan, 2003–2019.

		Population Allocation			Area Allocation		
		Beds	Health Technicians	Licensed (Assistant) Physicians	Beds	Health Technicians	Licensed (Assistant) Physicians
2003	*T*	0.0413	0.0583	0.0461	0.5750	0.6418	0.6200
	*T_W_*	0.0143	0.0110	0.0057	0.0462	0.0314	0.0316
	*T_A_*	0.0270	0.0473	0.0404	0.5289	0.6103	0.5884
	*C_W_*	0.3455	0.1888	0.1234	0.0803	0.0489	0.0510
	*C_A_*	0.6545	0.8112	0.8766	0.9197	0.9511	0.9490
2007	*T*	0.0656	0.0734	0.0619	0.6524	0.6826	0.6705
	*T_W_*	0.0099	0.0123	0.0068	0.0247	0.0316	0.0281
	*T_A_*	0.0557	0.0611	0.0552	0.6277	0.6509	0.6424
	*C_W_*	0.1512	0.1679	0.1092	0.0378	0.0464	0.0419
	*C_A_*	0.8488	0.8321	0.8908	0.9622	0.9536	0.9581
2010	*T*	0.0691	0.0857	0.0680	0.6568	0.7166	0.6856
	*T_W_*	0.0097	0.0118	0.0081	0.0180	0.0289	0.0282
	*T_A_*	0.0594	0.0738	0.0599	0.6388	0.6877	0.6574
	*C_W_*	0.1404	0.1382	0.1190	0.0275	0.0404	0.0412
	*C_A_*	0.8596	0.8618	0.8810	0.9725	0.9596	0.9588
2013	*T*	0.0506	0.0665	0.0624	0.6334	0.6876	0.6799
	*T_W_*	0.0064	0.0095	0.0067	0.0281	0.0344	0.0306
	*T_A_*	0.0442	0.0570	0.0557	0.6053	0.6533	0.6493
	*C_W_*	0.1273	0.1427	0.1067	0.0444	0.0500	0.0450
	*C_A_*	0.8727	0.8573	0.8933	0.9556	0.9500	0.9550
2016	*T*	0.0526	0.0690	0.0584	0.6412	0.7022	0.6795
	*T_W_*	0.0083	0.0063	0.0043	0.0308	0.0303	0.0314
	*T_A_*	0.0443	0.0627	0.0541	0.6104	0.6718	0.6481
	*C_W_*	0.1582	0.0911	0.0744	0.0481	0.0432	0.0462
	*C_A_*	0.8418	0.9089	0.9256	0.9519	0.9568	0.9538
2019	*T*	0.0640	0.0650	0.0498	0.6725	0.6973	0.6622
	*T_W_*	0.0066	0.0053	0.0038	0.0207	0.0329	0.0381
	*T_A_*	0.0574	0.0597	0.0460	0.6519	0.6644	0.6241
	*C_W_*	0.1027	0.0810	0.0766	0.0307	0.0471	0.0575
	*C_A_*	0.8973	0.9190	0.9234	0.9693	0.9529	0.9425

## Data Availability

Not applicable.

## Appendix A

**Table A1:** Agglomeration degree of medical and health resources in districts and counties of Taiyuan, 2003–2019.

		Year	Yingze	Xinghualing	Xiaodian	Jiancaoping	Wanbailin	Jinyuan	Yangqu	Loufan	Qingxu	Gujiao
PAD		2003	8.88	6.68	3.40	2.47	3.53	1.33	0.15	0.19	1.05	0.28
		2007	8.71	6.55	3.79	2.48	3.57	1.37	0.14	0.19	0.98	0.27
		2010	8.54	6.60	4.01	2.41	3.53	1.33	0.14	0.19	0.98	0.27
		2013	8.46	6.65	4.02	2.31	3.53	1.31	0.14	0.19	1.01	0.27
		2016	8.68	6.67	4.03	2.22	3.52	1.32	0.14	0.19	1.03	0.26
		2019	8.54	6.64	4.29	2.13	3.54	1.37	0.14	0.18	1.02	0.25
Beds	HRAD	2003	14.13	9.69	2.53	2.02	3.53	1.49	0.11	0.07	0.25	0.25
		2007	17.20	10.34	2.46	1.71	3.44	0.51	0.09	0.07	0.27	0.23
		2010	16.82	10.84	2.85	1.76	3.01	0.41	0.12	0.05	0.26	0.21
		2013	14.69	10.27	3.99	1.81	2.98	0.76	0.10	0.05	0.25	0.18
		2016	14.93	10.20	4.15	1.78	2.87	0.81	0.11	0.05	0.22	0.16
		2019	14.54	11.48	4.09	1.52	3.05	0.40	0.10	0.05	0.23	0.15
	HRAD/PAD	2003	1.59	1.45	0.74	0.82	1.00	1.12	0.76	0.34	0.23	0.86
		2007	1.97	1.58	0.65	0.69	0.96	0.37	0.67	0.37	0.28	0.85
		2010	1.97	1.64	0.71	0.73	0.85	0.31	0.87	0.27	0.26	0.79
		2013	1.74	1.54	0.99	0.78	0.84	0.58	0.71	0.27	0.25	0.66
		2016	1.72	1.53	1.03	0.80	0.82	0.61	0.76	0.24	0.21	0.62
		2019	1.70	1.73	0.95	0.71	0.86	0.29	0.74	0.26	0.23	0.61
Health technicians	HRAD	2003	13.95	11.53	2.24	1.95	4.01	0.81	0.06	0.06	0.26	0.23
		2007	16.21	11.21	2.47	1.47	3.91	0.54	0.05	0.05	0.25	0.23
		2010	16.85	11.61	2.64	1.49	3.66	0.48	0.05	0.03	0.19	0.21
		2013	15.51	9.84	4.17	1.65	3.66	0.63	0.05	0.04	0.15	0.17
		2016	14.72	11.25	4.11	1.51	3.47	0.61	0.05	0.03	0.18	0.14
		2019	14.31	11.19	4.30	1.40	3.54	0.60	0.05	0.04	0.21	0.13
	HRAD/PAD	2003	1.57	1.73	0.66	0.79	1.14	0.61	0.43	0.29	0.24	0.80
		2007	1.86	1.71	0.65	0.59	1.09	0.39	0.39	0.27	0.25	0.85
		2010	1.97	1.76	0.66	0.62	1.04	0.36	0.34	0.18	0.20	0.79
		2013	1.83	1.48	1.04	0.72	1.03	0.48	0.33	0.20	0.15	0.63
		2016	1.70	1.69	1.02	0.68	0.99	0.46	0.36	0.18	0.17	0.54
		2019	1.68	1.69	1.00	0.66	1.00	0.44	0.38	0.20	0.21	0.54
Licensed (assistant) physicians	HRAD	2003	15.08	10.03	2.60	2.13	3.69	0.85	0.08	0.06	0.35	0.20
		2007	15.82	10.48	3.08	1.72	3.78	0.58	0.06	0.05	0.24	0.19
		2010	15.44	11.00	3.10	1.89	3.66	0.64	0.04	0.04	0.19	0.19
		2013	14.78	10.24	4.03	1.85	3.63	0.65	0.04	0.04	0.18	0.17
		2016	13.89	10.74	4.35	1.76	3.47	0.67	0.04	0.04	0.22	0.14
		2019	13.36	10.47	4.54	1.54	3.60	0.72	0.05	0.05	0.31	0.14
	HRAD/PAD	2003	1.70	1.50	0.76	0.86	1.05	0.63	0.55	0.28	0.33	0.72
		2007	1.82	1.60	0.81	0.69	1.06	0.43	0.42	0.28	0.25	0.70
		2010	1.81	1.67	0.77	0.79	1.03	0.48	0.31	0.21	0.19	0.72
		2013	1.75	1.54	1.00	0.80	1.03	0.50	0.28	0.24	0.17	0.62
		2016	1.60	1.61	1.08	0.79	0.99	0.51	0.30	0.24	0.22	0.53
		2019	1.57	1.58	1.06	0.72	1.02	0.53	0.34	0.27	0.30	0.56

**Table A2:** Value of influencing factors of medical and health resources allocation in Taiyuan.

**Influencing Factor**	**Yingze**			**Xinghualing**			**Xiaodian**		
	**Beds**	**Health Technicians**	**Licensed (Assistant) Physicians**	**Beds**	**Health Technicians**	**Licensed (Assistant) Physicians**	**Beds**	**Health Technicians**	**Licensed (Assistant) Physicians**
X_1_	0.656	0.656	0.651	0.604	0.603	0.605	0.706	0.709	0.708
X_2_	0.675	0.675	0.670	0.629	0.627	0.629	0.763	0.767	0.765
X_3_	0.923	0.922	0.938	0.944	0.958	0.949	0.987	0.977	0.983
X_4_	0.987	0.987	0.973	0.979	0.965	0.975	0.980	0.973	0.981
X_5_	0.702	0.702	0.697	0.720	0.716	0.720	0.791	0.795	0.793
X_6_	0.794	0.794	0.786	0.846	0.838	0.844	0.875	0.882	0.878
X_7_	0.989	0.988	0.990	0.995	0.984	0.994	0.988	0.986	0.992
X_8_	0.989	0.991	0.981	0.990	0.987	0.993	0.992	0.990	0.993
X_9_	0.977	0.976	0.989	0.982	0.993	0.987	0.981	0.974	0.979
X_10_	0.976	0.979	0.976	0.991	0.980	0.988	0.993	0.989	0.997
X_11_	0.985	0.984	0.993	0.991	0.994	0.996	0.990	0.983	0.991
X_12_	0.563	0.563	0.560	0.604	0.602	0.604	0.564	0.564	0.564
X_13_	0.963	0.962	0.979	0.983	0.982	0.984	0.989	0.990	0.988
X_14_	0.969	0.968	0.985	0.983	0.988	0.986	0.980	0.988	0.985
X_15_	0.927	0.930	0.917	0.978	0.968	0.975	0.893	0.900	0.897
X_16_	0.973	0.973	0.963	0.973	0.960	0.969	0.969	0.979	0.973
**Influencing Factor**	**Jiancaoping**			**Wanbailin**			**Jinyuan**		
	**Beds**	**Health Technicians**	**Licensed (Assistant) Physicians**	**Beds**	**Health Technicians**	**Licensed (Assistant) Physicians**	**Beds**	**Health Technicians**	**Licensed (Assistant) Physicians**
X_1_	0.663	0.657	0.662	0.622	0.623	0.625	0.840	0.858	0.861
X_2_	0.677	0.671	0.676	0.652	0.654	0.657	0.858	0.878	0.882
X_3_	0.971	0.969	0.975	0.976	0.978	0.976	0.972	0.970	0.971
X_4_	0.840	0.828	0.837	0.924	0.928	0.934	0.837	0.855	0.859
X_5_	0.613	0.608	0.613	0.640	0.641	0.644	0.730	0.740	0.742
X_6_	0.656	0.650	0.655	0.677	0.679	0.682	0.816	0.832	0.836
X_7_	0.967	0.951	0.961	0.949	0.957	0.970	0.932	0.958	0.964
X_8_	0.926	0.911	0.921	0.973	0.965	0.950	0.948	0.977	0.983
X_9_	0.982	0.988	0.984	0.983	0.986	0.989	0.956	0.985	0.990
X_10_	0.924	0.909	0.920	0.921	0.927	0.939	0.927	0.953	0.959
X_11_	0.953	0.937	0.948	0.961	0.969	0.983	0.913	0.938	0.943
X_12_	0.610	0.605	0.610	0.537	0.537	0.538	0.619	0.624	0.625
X_13_	0.973	0.970	0.975	0.947	0.952	0.954	0.915	0.940	0.946
X_14_	0.974	0.971	0.976	0.920	0.926	0.938	0.897	0.920	0.925
X_15_	0.835	0.823	0.831	0.922	0.928	0.940	0.851	0.870	0.873
X_16_	0.884	0.871	0.879	0.906	0.912	0.923	0.928	0.954	0.960
**Influencing Factor**	**Qingxu**			**Yangqu**			**Loufan**		
	**Beds**	**Health Technicians**	**Licensed (Assistant) Physicians**	**Beds**	**Health Technicians**	**Licensed (Assistant) Physicians**	**Beds**	**Health Technicians**	**Licensed (Assistant) Physicians**
X_1_	0.917	0.907	0.903	0.885	0.879	0.871	0.873	0.870	0.874
X_2_	0.926	0.916	0.911	0.890	0.884	0.876	0.883	0.879	0.883
X_3_	0.993	0.981	0.976	0.996	0.991	0.980	0.997	0.996	0.994
X_4_	0.988	0.989	0.989	0.987	0.978	0.967	0.963	0.958	0.964
X_5_	0.855	0.847	0.842	0.812	0.807	0.801	0.863	0.859	0.863
X_6_	0.913	0.903	0.899	0.856	0.850	0.843	0.854	0.850	0.854
X_7_	0.994	0.987	0.982	0.995	0.989	0.977	0.989	0.984	0.991
X_8_	0.984	0.995	0.991	0.989	0.996	0.988	0.995	0.996	0.992
X_9_	0.988	0.995	0.991	0.987	0.995	0.991	0.997	0.996	0.995
X_10_	0.983	0.972	0.967	0.982	0.973	0.962	0.975	0.970	0.977
X_11_	0.965	0.954	0.949	0.962	0.954	0.944	0.979	0.974	0.981
X_12_	0.877	0.868	0.863	0.837	0.832	0.825	0.871	0.867	0.871
X_13_	0.990	0.993	0.988	0.989	0.985	0.973	0.939	0.935	0.940
X_14_	0.995	0.992	0.988	0.987	0.978	0.967	0.987	0.982	0.989
X_15_	0.803	0.795	0.790	0.788	0.783	0.777	0.799	0.795	0.798
X_16_	0.995	0.986	0.981	0.984	0.976	0.964	0.978	0.973	0.979
**Influencing Factor**	**Gujiao**								
	**Beds**	**Health Technicians**	**Licensed (Assistant) Physicians**						
X_1_	0.960	0.961	0.964						
X_2_	0.965	0.963	0.965						
X_3_	0.967	0.964	0.957						
X_4_	0.814	0.819	0.819						
X_5_	0.620	0.624	0.621						
X_6_	0.729	0.734	0.732						
X_7_	0.957	0.959	0.967						
X_8_	0.952	0.955	0.961						
X_9_	0.986	0.978	0.981						
X_10_	0.884	0.888	0.891						
X_11_	0.963	0.965	0.972						
X_12_	0.687	0.691	0.689						
X_13_	0.939	0.946	0.947						
X_14_	0.952	0.956	0.962						
X_15_	0.682	0.688	0.685						
X_16_	0.936	0.940	0.944						
